# How the care workforce navigates the digital ‘skills gap’: problems and opportunities from policy to practice

**DOI:** 10.3389/fsoc.2025.1552672

**Published:** 2025-04-24

**Authors:** Grace Whitfield, Erika Kispeter, Kate Hamblin, Diane Burns

**Affiliations:** ^1^ESRC Centre for Care, CIRCLE (Centre for International Research on Care, Labour and Equalities), University of Sheffield, Sheffield, United Kingdom; ^2^ESRC Centre for Care, Department of Health Services Research and Policy, London School of Hygiene & Tropical Medicine, London, United Kingdom; ^3^Sheffield University Management School, University of Sheffield, Sheffield, United Kingdom

**Keywords:** care workforce, technology, digital, skills, England, policy

## Abstract

**Introduction:**

Care systems and services across the globe are under pressure, with challenges related to the recruitment and retention of the care workforce identified as a particular issue. In England, digital technologies are presented in policy discourse and strategy as a potential way to navigate these complexities by delivering faster, cheaper and better care. The workforce, meanwhile, tends to be defined as requiring better digital skills to enable the full potential of digital technologies to be realised.

**Methods:**

We carried out qualitative case study research of seven social care provider organisations, involving interviews with a total of 62 people from a range of roles across the care workforce and observations of work-based practices. Drawing on this data, we explore in-depth the workforce’s experiences of and perspectives on using new technologies, and the requisite skills.

**Results:**

The results show how the issue of maximising the adoption of technologies is (1) affected less by a deficit in worker skills, and more by the type of digital technologies in use, the job role of the worker, and the type of care provider, (2) can be facilitated by a supportive learning environment, and (3) can be impeded by issues in the functionality of systems and devices.

**Discussion:**

We show a disconnect between the assumptions made in policy discourse and the practicalities and variations in how workers adapt, apply, and develop skills. We also explore the importance of peer support, albeit hindered by time constraints and sometimes overly relying on individual workers. In addition, the paper highlights the importance of understanding how new technology adoption can be stymied by the design of the technology itself, rather than the result of the workforce’s lack of digital skills *per se*. An unintended consequence of defining the problem as a skills mismatch and the solution as skilling the workforce is that the abilities of the workforce to creatively and flexibly manage the short-comings of digital devices and systems are overlooked and under-utilised - reflecting a wider failure to acknowledge and compensate care workers’ skills.

## 1 Introduction

The adult social care (ASC) sector in England has, as with care sectors globally (United Nations, 2018), struggled with underfunding, increased demand, and decreased workforce supply. Across the four United Kingdom (UK) nations these issues represent the effects of fragmentation, privatisation, and market instability instigated in the 1980s (Rubery et al., 2015). They are issues exacerbated in recent years due to ‘Brexit’, the COVID-19 pandemic, and an ageing population (Turnpenny and Hussein, 2022). Increasingly, technology has been seen as a means of resolving pressures on the sector by delivering faster, cheaper and better care. The ‘technological fix’ has been a key feature of policy discourse globally (European Commission, 2018; World Health Organization, 2021), including in England (Eccles, 2021; Whitfield and Hamblin, 2024). The English Government has published a flurry of policy documents advocating the use of technology in care since the early 2000s (Whitfield and Hamblin, 2022; Wright and Hamblin, 2023). The types of technology referenced in policy documents vary, including digital social care records (Department for Health and Social Care, 2022a), acoustic monitoring (Department for Health and Social Care, 2021), mainstream devices such as smart speakers (Department for Health and Social Care, 2021) and ‘wearables’ (Barclay, 2022), and Artificial Intelligence (AI) to detect issues such as falls (Javid, 2022), as well as the use of digital to enhance flows of data and information both within social care and across into the health sector (Department for Health and Social Care, 2022a). There has, however, been a slower adoption of technologies than hoped for in policy aims - as well as more mixed outcomes than expected (Hirani et al., 2014; Glasby et al., 2021; Eccles, 2021). These issues are often linked in Government strategy to a lack of digital skills among the care workforce, with workers needing to upskill, and take part in formal or informal training. However, the perception of a digital-skills deficit can ‘overplay the complexity of the new skill demands’ (Lloyd and Payne, 2023: 1085). In this paper, we use case studies of seven care providers to understand existing skill levels and skill development in the sector. In doing so, we discuss whether workers’ skill levels impede the transformative potential of technology and disrupt the ‘triple-win’ discourse (Neven and Peine, 2017) in policy and practice space, which asserts that technology is good for the economy, good for people receiving care, and good for society.

### 1.1 Definitions of digital skills

According to the policy paper ‘Data Saves Lives’ (Department for Health and Social Care, 2022a), digital skills are ‘necessary in all roles and at all levels’. Another policy paper, ‘A Plan for Digital Health and Social Care’ (Department for Health and Social Care, 2022b), includes an aim to ‘ensure our health and social care workforce have the right skills to apply these technologies successfully and our organisations have cultures that foster innovation’. In these key policy documents, however, skills in relation to digital technologies are not clearly defined, for example extending into areas such as cyber security and cyber ‘hygiene’. Skill development is also understood broadly - the long-awaited White Paper ‘People at the Heart of Care’ (Department for Health and Social Care, 2021) emphasises ‘setting competencies reviewing the skills and knowledge required, putting in place career paths, and creating communities of practice to rebuild a credible and self-respecting profession’. This lack of specificity was an issue highlighted 10 years ago in the review commissioned by Skills for Care, the strategic workforce development and planning body for ASC in England (Kispeter, 2018: 16). It reflects wider debates in how ‘digital skills’, and indeed ‘skills’ are defined and understood (Green, 2011). In literature, ‘skill’ has been conceptualised as having and being able to apply vocational knowledge - acquired through a mixture of formal and on-the-job learning - but it can also be understood as referring to expectations of employers for their workers. Other approaches see skill as not only what employees ‘have’ but also how they use skills at work (Warhurst and Luchinskaya, 2018), or highlight how employers can enable workers to use their existing skills to improve performance (Appelbaum et al., 2000). What is regarded as a skill is thus contextual, can change over time (Grugulis et al., 2004; Keep and Payne, 2004; Warhurst et al., 2017), and relates to other factors such as capability, adaptability, and confidence (Keep and Payne, 2004).

Skills involved in utilising digital devices and systems require particular ‘contextualising’ because digital tools and the skills needed to use them at work change rapidly, i.e., digital technologies quickly become obsolete. This illuminates the need for non-technical skills, such as literacy and numeracy, to enable workers to adapt to fast-changing technologies (Nedelkoska and Quintini, 2018). The concept of digital skills is also argued to be ‘fuzzy’ as it is used in ways that overlap with related concepts, such as digital competencies, capabilities and literacies (Kispeter, 2018). For example, UNESCO Institute for Statistics (2018: 138) refers to career-related competences as part of digital literacy, and describes such competences as ‘the knowledge and skills required to operate specialized hardware/software… or the use of learning management systems to deliver fully online or blended courses’. In response to calls to address vague and obsolete definitions, Orlik (2018), recommends definitions of digital skill that are sufficiently broad to identify the group of people who need the skills, the place - context in which they need to use the skills - and the period, that is, the timeframe in which these skills are relevant.

Different definitions of digital skills have been drawn upon to inform various digital skills/capabilities frameworks (e.g., Vuorikari et al., 2022; World Economic Forum, 2020). As proposed in the ‘Data Saves Lives’ (Department for Health and Social Care, 2022a) paper, the ‘Adult Social Care Digital Skills Framework’ was developed by Digitising Social Care (a programme funded by the Department of Health and Social Care) in 2023 in collaboration with Skills for Care. This framework applies to the context of England, with other frameworks also developed in Scotland and Northern Ireland (Department of Health and Care Northern Ireland, 2022; NHS Education Scotland, 2024) and an assessment tool in Wales (Social Care Wales, 2024). The English framework categorises digital skills into seven domains (e.g., ‘using technology’ and ‘using and managing data’), and describes two levels of increasing proficiency within each domain. It contains both wide ranging and general knowledge criteria and specific skill statements aligned with particular care activities (e.g., ‘Use technology to help people to build and maintain relationships and participate in their community’), and specific types of care-focused technologies [digital social care records, electronic medication administration systems (e-MAR)], as well as fundamental skills [e.g., ‘Connect to the internet using the Wi-Fi settings and enter the Wi-Fi password when required’ and ‘Turn on a device and enter any information required (e.g., usernames and passwords) to safely login’]. The types of skills addressed in the framework are therefore wide-ranging, with a number of different intended audiences including those working in social care, employers, local authorities and Integrated Care Systems [ICSs- partnerships that bring together National Health Service (NHS) organisations, local authorities and others], and learning providers. Yet a key challenge in frameworks such as this remains in understanding the range of different devices and systems in use across the care sector and within its varying contexts.

### 1.2 Levels of digital skills

The skill levels of the workforce are presented as part of the ‘policy problem’. The White Paper referred to in the previous subsection, for example, states: ‘our ambitions for a digitally enabled care system cannot be realised without a workforce that is skilled and confident in the use of technology’ (Department for Health and Social Care, 2021). Such statements reiterate a common trope from the policy discourse whereby lack of skills and confidence are conflated and assumed to create barriers to using digital technologies. This policy discourse also constructs the workforce as homogeneous (Hamblin et al., 2023), underplaying the diversity of roles across the adult social care sector. In 2023/4, around 1.59 million people worked in the adult social care sector in England, employed across 18,500 organisations. Across this workforce, the roles are highly varied, ranging from care workers (905,000), managers (123,000), administrative staff (46,000), Occupational Therapists (23,000) and registered nurses (34,000) (Skills for Care, 2024), with differing associated levels and types of skill. According to Blake et al. (2021), self-assessed levels of skill vary a great deal across roles: staff who assessed their skill level as very low were typically older care workers and nurses, and a third of registered managers reported gaps in the digital skills of frontline staff. The authors also highlighted that confidence and skills were closely linked to opportunities to use digital technology at work, including such basics as having internet access. Yet differences in opportunities to use technologies vary. Some potentially larger employers with more resources lead in the adoption of digital technologies while small and micro providers are at the trailing edge. As noted above, context also matters in that employers can enable workers to better use their existing skills to improve performance (Warhurst and Luchinskaya, 2018), through creating an environment that provides opportunities to learn and use digital skills and motivates workers. Policy documents which focus entirely on care workers and propose upskilling risk ignoring this workplace context and its effects on determining skill levels and opportunities for their use.

### 1.3 Skill development

In approaches to ‘upskilling’, policy documents have largely focused on digital skills training. Sometimes - as noted above - skill is also conflated with building confidence in the use of technology. For instance, the ‘Data Saves Lives’ (Department for Health and Social Care, 2022a) paper describes the importance of ‘an inclusive approach to training opportunities to improve the data and digital literacy of the adult social care workforce’. Strategies for addressing the skills gap referenced in the 2021 White Paper (Department for Health and Social Care, 2021) are ‘targeted digital leadership’ to enact cultural change, and a ‘comprehensive digital learning offer’ with online training and resources. Types and approaches to training can be determined by its status as statutory, mandatory, additional or developmental. There is statutory training in the sector, related to specific legislation [e.g., the Health and Safety at Work Act 1974 (Health and Safety Executive, 1974) and the Management of Health and Safety at Work Regulations 1999 (Health and Safety Executive, 1999)] and mandatory training - deemed essential in the commissioning and contracting of social care provision and compulsory for service provider organisations to formally arrange for their workforce. Currently the ‘Adult Social Care Digital Skills Framework’ (Digitising Social Care, 2023) and training related to aligned areas such as data protection (GDPR) is classed as ‘additional training’, and therefore ‘[d]efined by the employer based on the needs of the service and people who draw on care and support’ (Skills for Care, 2024: 9). Digital skills are not included in the Care Certificate, a 12-week induction training, specifying a set of standards for care workers (Skills for Care, n.d.) but are highlighted as a key learning and development priority in the new Level 2 qualification for care workers (Department for Health and Social Care, 2024a).

The success of formal training programmes or opportunities, however, can be mediated by factors internal and external to the employing care provider organisation. These factors have been identified in the context of digital transformation projects in nursing homes in the United States of America: Avgar et al. (2018) illustrate how the homes’ work practices, workplace culture, peer support, and broader employment relations had a strong influence on the success of implementing the electronic medical records systems. Approaches drawing on more informal mechanisms for skill development, for example support provided by ‘Digital Champions’ and on-the-job training involving co-workers, was shown to be an important factor in acquiring digital skill (Lloyd and Payne, 2023). Blake et al. (2021) also found that without tailored peer support, care workers reported higher levels of stress and anxiety when using formal digital technology training, and additionally, that limited opportunity to use digital technology day-to-day can pose a further barrier to developing and embedding skills. Specific to domiciliary care contexts an evaluation study of pilot projects implementing new technologies emphasised that digital skill development should connect to pay and career progression (Oung et al., 2021). At an operational level research studies in both health and social care sectors found managers to expect digital change to occur without accounting for adequate time investment for frontline staff to adapt to using digital technologies (Kaihlanen et al., 2023; Maguire et al., 2018).

Across policy claims and strategies related to ASC and digital skill development, the range of potentially diverse technologies being deployed within care provider agencies, numerous ways workers may interact with them (Højlund and Villadsen, 2020) and varied implications for identifying skill level, gaps and development needs are, we argue, largely unknown (Hamblin, 2022). This paper is focused on addressing these gaps in understanding through three questions: what digital technologies and associated skills are social care workers in England using in practice? What digital skills are present in the care workforce, and how do these align with the devices and systems in use? How are digital skills being developed at the care provider level?

## 2 Materials and methods

### 2.1 Design and data collection

This paper draws on data gathered through seven in-depth qualitative case studies of care provider organisations in England, collected during 2023–2024. Our sampling strategy considered a number of factors. First was the type of care provider. We aimed to research a mix of domiciliary care, residential care (including supported living), and support services like day care to provide insight into different dimensions of the care workforce. Our sample included providers operating on both a for-profit and not-for-profit basis, varied in size and in the type of care they delivered to people using the services. The smallest organisation was Clover (all names are pseudonyms), with 50 staff members and 56 people they support, and the largest was the care trust which Anise and Dill belonged to, which comprised 4,800 staff and 3,500 residents overall. The second factor was the technology focus of the organisation. We considered whether our sampling allowed sufficient insight into the range of technologies used in the sector [described in a working paper (Whitfield and Hamblin, 2022) as technologies used to assist, monitor, organise and record care, collect and analyse data, and connect people]. We also looked at whether providers claim to be innovative and ‘tech-forward’, and whether there are particular technology-impacting obstacles (e.g., rurality and the impact in 4G/ 5G signal). The third factor was the location of the care provider. To provide insight into the care workforce and technology use across England, we looked for providers across different geographical regions. Some of the providers selected are situated in one place, while some are more spread out. Varying the location enables analysis of care within different economic contexts - and different levels of reliance on local authority packages - and varied population characteristics, such as levels of migration, diversity of ethnicity, and ageing. In total, we contacted 15 care providers, selecting a total of seven organisations for case study. Table 1 describes the characteristics of the provider organisations, e.g., funding arrangement, size, type of provider, and location, and the number of interviews, hours of observation and documents collated at each site. Each provider is also allocated a pseudonym.

**Table 1:** Case study and fieldwork information.

Care provider pseudonym	Brief description	Fieldwork conducted
Anise	Residential care setting in the south of England; mix of self- and local-authority funded placements; part of a wider trust of residential care settings across the south of England. Not-for-profit.	Observational field notes; analysis of marketing materials; 12 interviews (6 care workers, 2 care leaders, 1 manager, 1 deputy manager, 1 administrator, 1 housekeeper).
Basil	Domiciliary care setting in the north of England; primarily local-authority funded users. For-profit provider, employee owned as of 2024.	Observational field notes; analysis of marketing materials; 6 interviews (1 care lead, 1 director, 1 locality manager, 1 manager, 1 administrator, 1 training and compliance manager). Observation of training session (lasting 1 h) and group interview with 8 care workers (lasting 30 min).
Clover	Supported living, day care and outreach services for adults living with learning disabilities in the Midlands; primarily local-authority funded users. Not-for-profit (PLC).	Observational field notes; analysis of CQC report; 7 interviews (2 care workers, 2 team leaders, the training manager, the digital lead, and the CEO).
Dill	Residential care setting in the south of England; self-funded placements only; part of the same trust of residential care settings across the south of England as Anise. Not-for-profit.	Observational field notes; analysis of marketing materials; 8 interviews (3 care workers 2 care leaders (1 on nights only), 1 manager, 1 deputy manager, 1 tech lead).
Elderflower	Residential care setting for older people (including nursing and specialist dementia care) in the south of England; self-funded residents and local authority placements.	Observational field notes; analysis of marketing materials; 11 interviews (2 health care assistants, senior health care assistant, registered nurse, dietician, head chef, pharmacy technician, head of therapies, digital care lead, IT manager, nursing manager, director of research and innovation).
Fennel	Domiciliary provider in the south of England; mix of local-authority and privately funded users; has a supported living provider attached. For-profit.	Observational field notes; analysis of marketing materials; 11 interviews (1 broker (between care provider and local authority), 4 care workers, 1 care coordinator, 2 recruitment/ HR staff, 1 compliance and quality staff member, 1 manager).
Ginseng	Domiciliary provider in the north of England; mix of local-authority and privately funded users; organisation has multiple branches across regions. For-profit - separate residential division has involvement from real estate investment trusts.	Observational field notes; analysis of marketing materials; 7 interviews (1 care worker, 1 care coordinator, 1 operations director, 1 HR recruiter, 1 administrator, 1 manager, 1 branch trainer).

For the focus of this paper we draw mainly on data gathered through the interviews with staff from across different job roles in all seven case study sites. All research participants were provided with information sheets and consent forms prior to engaging in the project. To ensure their informed consent was obtained to take part in the research, participants were given the opportunity to ask questions about the project and voluntary participation was emphasised, as was confidentiality; all care provider names used in this paper are pseudonyms. The study received ethical approval from both the University of Sheffield Research Ethics Committee (reference number: 052354) and the London School of Hygiene and Tropical Medicine (reference number: 29579), and University of Sheffield research governance sponsorship (dated 07/08/2023, project number: 181380) and followed relevant local authority research governance procedures where applicable.

### 2.2 Analysis

Comparative data gathered from each case study were triangulated using a complex approach of method and data, and investigator triangulation (Denzin, 2009). Understanding skills through the perceptions of interviewees from just one group within the workplace is potentially limiting: Lloyd and Payne (2023: 1090) note that ‘asking a manager about the skills of a care worker assumes that the managers understand what is involved, while job holders may underrate practised skills or ‘tacit’ knowledge that can be hard to tell’. We therefore mitigated this subjectivity of perspectives by interviewing both managers and care workers (including senior care workers or ‘team leaders) and triangulated the data collected from different perspectives. Other workers interviewed were cleaning and maintenance workers, team leaders, nurses, quality assurance staff, HR staff, a head chef and pharmacists. For the in-depth interviews, separate topic guides were created for those in management, administrative and care worker roles (including anyone directly providing care, such as housekeeping staff in some residential care settings). The topic guides for those in management roles included questions related to the digital technologies in use (including rationale for their selection; benefits and costs; issues with usability; abandoned technologies) and skills (including those required to use the digital technologies; the skill level of their workforce; and training and other strategies to develop skills). For care workers and others directly providing care support, the topic guides included questions again on the digital technologies (including what devices and systems they use on an average shift; ease of use; benefits and drawbacks) and skills (including self-rated digital skills, reflections on skills training and development).

We further diversified the data using additional methods - carrying out a group interview with care workers, observing training sessions, making observational field notes, and examining publicly available documents about the organisation. The latter method utilised a situational analysis approach (Clarke, 2021) to provide detail about the externalities of the organisations. We also used investigator triangulation in the data collection at case studies sites (GW case studies Basil, Fennel and Ginseng; EK case studies Clover and Elderflower; KH case studies Anise and Dill), and in the analysis of the data, with all data coded independently by three authors (GW, EK and KH) to facilitate inter-coder reliability. The NVivo software tool was used to enable ease of comparison, with coding frameworks and the coded data cross-checked; the coding framework was then discussed and refined, with the wider team reflecting on the findings as related to our research questions, and in turn the wider literature on digital technologies, care and skills. We took an abductive approach to the triangulation analysis of the data (Timmermans and Tavory, 2012). We first coded the data deductively, with codes drawn from topic guides based on the research questions designed to explore issues related to our research aims and questions (as set out above), and then inductively, to produce second-level codes to draw out additional complexities related to digital technologies in use at the case study sites, skills and skill-development. For the first author the initial codes were: champions (‘super users’), peer support, lack of skills, recruitment/retention, personalised training and support, skills, training. The second author listed: adapting to tech (age, ease of use), developing skills (upskilling, recognition), existing skill levels (pre-employment), recruitment (onboarding, retention), training methods (online, face to face), and training on tech (on apps, CMS). The codes of the third author were: training, support, skills, previous experience of technology and care, learning on the job, intuitive, existing skills or experience with technology, apprehension or excitement, and ‘champion’ model.

The codes are grouped into themes, as laid out below. The first theme (subsection 3.1) relates to Question 1 and Question 2. It explores the types of technology used in practice and workers’ and managers’ perceptions of the skill required to use technologies - their adaptability, and differences across groups. The second theme (subsection 3.2) answers Question 3 - what facilitates the development of skill, and what obstructs that development? The third theme (in subsection 3.3) was identified through the analysis of the data: that care workers and managers often view the limitations of digital systems and devices used in social care in ways that are disconnected to skills development. By comparing across case studies in these subsections, we were able to corroborate findings and draw out areas of similarity across provider types and workers’ views and experiences and indicate areas of difference - as discussed in the final section.

## 3 Results

### 3.1 ‘I’m computer literate’: technologies used and existing workforce skills

The technologies used by the case study organisations were heterogeneous; Table 2 below provides an illustration of the variety of technologies across areas of recording and rostering care, assisting care, monitoring care, and those used in office systems.

**Table 2:** Types of technology in use at case study sites.

Case study	Recording care and rostering	Assisting care	Monitoring technologies	Office systems
Anise	Digital care records and rostering on the same system.	Hoists and stand aids - mainly used on one floor, where the residents who had mobility issues were living.		Office staff had a separate online system for recruitment, where CVs and applications were stored. Personnel files had recently been scanned and uploaded, and pay slips had been digitised.
Basil	EMAR; digital Care Record with GPS, electronic rostering digital login via QR codes.	Hoists, stand aids, PEG feeding.	Telecare - pendant alarms, wrist pendants, and fall mats - connecting to Alarm Receiving Centre (ARC). Sensors and monitoring equipment were put in place by family members of those using care, such as Ring doorbells - sensor equipment in people’s homes ‘sporadic and not very well coordinated’ (manager).	Online portal for payments from local authority and direct payments via CCG, digitised recruitment forms and applications, small amount of online training, app for staff to receive wages early.
Clover	Used digital care record and rota system but only when it was a requirement from the local authority, then returned to paper records and Excel.	Laptops, iPads and smart TVs in assisted living facilities. Staff help individuals to identify assistive technologies.		Use of Microsoft 365, moving away from WhatsApp (to Viva Engage) and from MS Teams channels to a newly developed intranet to share files. In-house developments: data entered into Microsoft Forms is automatically fed into Excel tables. Digital training platform and digital payslips. QR codes help staff to find shared documents and enter data instead of using paper forms.
Dill	Online ‘e-care’ digital records and rostering system.	Beds that can be positioned at floor level and tilt, fundraising for ‘giant iPads’ to use for sensory games.	Acoustic monitoring system: alerts would be monitored during the night by a care lead. Call bells used during the day.	Office uses PC laptops, acoustic monitoring computer which can generate pie charts of data, Teams meetings with Care Leads, personnel files both paper and digitised.
Elderflower	Used digital care planning, eMAR, an HR/payroll system for clocking in/out. They were upgrading the digital care planning system (devices and log-in) and introducing a new eMAR.	Used manual hoists and digital hoists, operated by remote control, digital weighing scale wheelchairs and beds, and a variety of digital equipment for physiotherapy.	Used sensors for monitoring residents. Acoustic monitoring had been trialled but abandoned - the sensitivity of the system made it unsuitable for a care home environment. Used a call bell system linked to sensors instead.	Online training platform, log in and out for visitors, a system for logging maintenance tasks. At the time of fieldwork, they were developing the intranet.
Fennel	Digital rostering separate to the system for support plans and care records with the apps ‘talking to’ each other.	Hoists, Alexa for medication reminders, had been part of a local authority trial giving out iPads.	Pendant alarms, call bell systems, and fall mats. Had trialled more extensive sensor equipment which would ‘show any time they boiled the kettle, opened the fridge, maybe turned the oven or microwave’ - the trial was stopped.	Recruitment processes and onboarding still ‘oldschool’ - ‘we have got spreadsheets’. In the process of switching to another platform which would provide all these functions. ‘Support planning’ team which audited care records app.
Ginseng	Digital care records, rostering, care planning, and medication requests.	Tracking hoists, and floor standing hoists.	Pendant alarms, wrist pendants, and fall mats. Some family members had installed cameras and Ring Doorbells. Sensor alarms on doors. Currently trialling a sensor system with local authority.	Staff app with support and awards, Microsoft package.

The skills involved in using the technologies also varied. Skills required when using digital care management systems (for care planning and care records) were: familiarity with typing and navigating touch screens, logging in and out of devices, entering and accessing data, taking photos and attaching them to records, accessing digital ‘forms’ using a QR code and reading and sending emails, including to the families of the people they supported. Some more senior staff would also edit care plans, locate archived information in digital care records, and share information with local authorities and regulators. Staff also had to learn how the particular systems and hand-held devices worked. According to the manager at Basil, a difference in skill level between staff in different roles was evident, but not in ways characterised in the policy discourse. He argued that ‘our regulators or our commissioners are less IT savvy’ than direct care workers, and went on:

*How bizarre that in our world that we live in that our 100 carers with poor academic background, the cost of living crisis and all the crap that’s going on in their life, not being IT savvy. Now all of a sudden they’re on their phones working a digital app, taking photos of medication, sending them across and to think we’ve got all this brilliant stuff. And then our very well trained academic people that come in to inspect or audit and evaluate, struggle immensely with it.*

The emphasis on a digital skills gap referred to earlier did not, from his perspective, apply for care workers; it did, though, apply for others in the social care system.

Care staff adapting to newly introduced technologies did not appear to be understood by interviewees as an explicit upskilling. When asked about whether staff were developing their skills, the trainer at Ginseng said ‘I just generally think that most people, these days, can use a phone-based app. So, the upskilling, is that a requirement? Probably not. Probably not, but then again, if they went on to work in a nursing home where a drugs round is on a laptop, then that’s different’. A care worker at Fennel - when asked the same question - said: ‘it depends, because actually (laughs) how should I put it? I’m computer literate’. The worker went on to say that the ‘new’ skills she had learnt were those related to assistive technology, like hoists.

This ease with technology was, however, impacted by key factors discussed in this section such as prior experience using smartphones. A care worker at Fennel said: ‘I think if you are somebody that has got a mobile phone or an iPad, and you go on Facebook or TikTok or WhatsApp or any of those things, or you go onto anything, on to the web and you have a look, you are used to pressing buttons, ticking boxes’. Yet interviewees who referred to the usability of the platforms did describe some initial trepidation about the new systems when they were first introduced. At Anise, the Care Lead said that ‘we have some staff who do not use smartphones so they were very apprehensive, they were unsure’. Others discussed this adaptability from personal devices to the digital systems as more nuanced. A manager at Fennel referred to ‘people that use smartphones every day, probably on their social media constantly, but have needed more support with the app’. The advantage which being comfortable with using mobile phones gave in adapting to the digital systems was also mediated by the type of phone. The Training Manager at Clover told us that her brain needs to adjust every time she switches from her personal iPhone to her Android work phone: ‘I said to somebody, “I’ve got a big black dot on my work phone,” and he goes, “No, that’s the camera.”’

Interviewees also referred to a perception that older workers would struggle more with the skills involved in using the apps, but said that this was not always the case. Instead older workers - who might initially express some ‘resistance’ - could soon become adept at using the systems. A housekeeper and care worker at Anise said:

*They’re not very good on the technology, the older generation … one lady, she’s still got a 3410 mobile [i.e. not a smartphone] she can’t use. She was panicking and now she’s one of the best ones using it and she’s like, “I don’t know why I panicked, I like using it.” And yeah, she’s really good at it.*

English language skills were another factor among workers’ existing skill levels with using technology. A manager at Ginseng referred to ‘sponsorship’ staff - those on a Health and Care Worker Visas (a skilled worker visa route for migrant workers to enter the UK for work) - as struggling with the app. At Elderflower, a team leader described how some staff members who had English as their second language found adding therapy notes to care records difficult. However, like older workers, these staff members were able to adapt over time: ‘I can think of one member of the team who came over to us, initially was just getting by, over a period of a year his vocabulary really improved and now I can see he’s more willing to use technology’. Confidence in their language skills was also an issue for domestic workers. An interviewee at Anise - employed as a housekeeper and care worker who described themself as ‘quite techie’ - was still apprehensive at first:

*I’ve grown up with a phone and that, but something like that I was a bit nervous because obviously everyone can see what you’re writing … But then you get more comfortable with it, because like you’re thinking it’s just like being on my phone, it’s like sending a text or something.*

Disabilities and specific learning difficulties, such as dyslexia also made it more difficult to adapt technologies. A staff member at Elderflower explained how their visual and hearing impairment made using small screens challenging: ‘You have to scan, you have to take photographs, you have to do lots of things. On a small device I could not see it and I felt really frustrated if I’m honest’.

Across the organisations, different groups were highlighted as taking longer - but there seemed to be a consensus that people would get there in the end. As an office worker at Ginseng described it, ‘people definitely struggle with the e-learning, some people struggle more with the work phones, but once you get the hang of it it’s fine, it’s just it takes a different amount of time for each person’. Yet there were contrasts in the skill levels required to operate some of the other types of technology described in Table 2, meaning that adapting could take longer or require more skill development (as discussed below). For example, at Clover, the Digital Lead said of the Microsoft 365 software:

*We've got lots of staff on different levels of understanding, say, for example SharePoint or the Teams channels. It's becoming clear that we've a whole range of experience … and that's for frontline staff and for the head office staff, which proves challenging, especially because both sets of people need very different levels of understanding of all the things that we use.*

A team leader at Elderflower referred to Excel as particularly challenging: ‘If I gave an Excel sheet to one of our team members and asked them to update the equipment inventory, it would come back to me on a piece of paper, so I think not everyone feels as comfortable as each other’. Familiarity with Microsoft 365 software was part of the selection process for therapies staff, however, it was not only the job applicant’s digital skills they were interested in, rather their ability to present ideas effectively using digital tools:

*I often say, ‘can you write me just a one page of why you’re applying for this job’. And actually that is often more useful to me to get an idea of how people manage technology. You could see, for example, what programme they’ve used, how they format it, can they pull it together, can they type, things like that.*

Interviewees at Ginseng and Fennel said that technology was not something which was discussed much through their recruitment processes, but as a lot of job advertising was done through social media, applicants were often likely to have some familiarity with technology. A manager at Ginseng said ‘technology plays a part because we have online application forms, we use a recruitment platform’. Some applicants would complete these forms in the office: ‘we’ll do it alongside them, that helps us because then we are able to work out how much more support they are going to need if they were taken on, to do the technology side for the apps’. Unlike for therapies staff at Elderflower, the lack of familiarity or confidence did not mean that applicants for care worker positions were a write off; rather, they might need more of the support discussed in the below section.

### 3.2 ‘We’ll do it online and we’ll do it together’: facilitators to skill development

Across the organisations, some level of formal training in digital skills would be provided for new staff members, and when a new technology was first adopted by the organisation. Sometimes this was online and sometimes it was in person. For example, when Elderflower first started to use digital care planning, in-person formal training was provided by the technology company. During the Covid-19 pandemic training became more ‘word-of-mouth, “this is how you use it”’ (Elderflower, dietician), but when the technology company developed online training, it became part of the induction for new staff. The super user, who was involved in introducing the care planning system at Elderflower argued that formal training was necessary: ‘I pushed for the online training, because we aren’t using the system optimally, because people were not getting trained’ but acknowledged that ‘it’s probably not the best training, but it’s helping to fill a gap at least’. Indeed, some care workers found the training difficult and had to retake the test to complete their induction.

The amount of formal training on using the care management systems was described in contrasting ways by staff within the organisations. At Anise, a care worker referred to online training to use the record system app, which lasted ‘about an hour’. Another care worker referred to more hands-on training too, saying ‘I know the trainer was around for a few days and able to answer questions as well’. According to one manager, the senior staff team and care leaders all had additional training over three dates, while another manager described the additional care planning training as 3 hours, and said, ‘the training for everything except the care plans was good’. Similarly, there were different accounts of training from interviewees at Dill. A care worker recalled, ‘I think there was an e-Care person that came in. I cannot remember where she was from, but she seemed to know what she was on about’. A second care worker said, ‘I know colleagues who have done the training, I have not done it’; a third said, ‘I do not think I had any official training on it that I can remember’.

At Clover, where they had stopped using electronic monitoring of care visits (but had previously received formal training on it), internal training on using Microsoft 365 software was offered to those who needed help. One of the team leaders, who did not need support, had developed their own skills in their free time: ‘I’ve learnt to use Excel, I’ve learnt that by watching YouTube videos, by looking up online how to do certain things’. The training to use remote monitoring systems was also minimal across the organisations. A care worker at Dill referred to a lack of training on using the emergency bells in residents’ rooms: ‘for me, it took six to 8 weeks until I had the training. Luckily, we did not need to use it but it’s something that should be taught on the first day that you start’. The organisation had received some training for the acoustic monitoring system, provided by the technology company: ‘I think they are quite a small company … they are online, as I said, 24/7 and they really want to get it right for us’. At Fennel, staff had not had training on how to use the devices which connect to an ARC function. A care worker was of the opinion that ‘it would be useful to be like, “This is what [the technology] looks like. This is what…” I’d never seen that’ and said that when it was alerted ‘I was like, “What is that noise?”’.

Alongside, or instead of, formal training on the different technologies ran a model of learning and training via ‘Digital Champions’, where individuals would be nominated or volunteer to provide additional digital support for their colleagues. This model (Skills for Care, n.d.), was used at Anise, Clover and Dill. Elderflower had a similar ‘super user’ system, which they were reorganising, using Skills for Care’s training materials. While prior knowledge among workers of using technology was utilised, there was seemingly not an increase in reward for Digital Champions across the organisations. There were also some issues with the sustainability of the champion model in its reliance on individuals. The manager at Anise, talking about a member of the maintenance rather than care staff, who was nonetheless ‘a Digital Champion’, said:

*If he’s on annual leave, oh, they struggle, because he’s really good. … In fact one of the residents this morning said, “My talking watch isn’t working.” So I had a look and I thought, yeah, I don’t know what’s the matter with it. It’s reading the right time, the clock hand’s going around, when you press the button it says it’s 12:00 am. So I said, “Well, he’s on holiday, I’m going to have to leave him a message”.*

The champion model was also often accompanied by other forms of peer support, of being shown how to complete tasks by colleagues. A care worker at Clover, who had not used iPads before they were introduced at work, recalled how colleagues helped them to get started: ‘I’ve just got them to show me and I’ve just written it down and copied it basically’.

‘Learning by doing’ was considered by interviewees to be more useful than formal training. At the domiciliary care companies in particular, it was in shadowing that a lot of the useful being shown how to use technology would occur. At Fennel a care worker said: ‘I think we were taught how to use it. We were taught, but we were not shown. I for one was not shown, but your first shadow shift, you would be shown by whoever you are shadowing’. The shadowing was more substantial and useful in part because during the initial training, workers would not be given full access to the data on the apps - this was because workers might drop out during the training stage. Instead, in those training sessions, ‘we get screenshots and they talk through things, but we do not actually get any live training’. The trainer at Ginseng also saw ‘shadowing’ as far more useful than training sessions, claiming: ‘it goes back to the old medical adage, does not it: see one, do one? I know it sounds really blunt, but part and parcel of the shadowing is learning how to use the app, because there’s little point in doing it here’. Shadowing was sometimes adopted as a way around the resource intensiveness of formal training. The manager at Basil said that training on digital tasks would be included as part of the induction for new starters, but said that digital training was not something that local authorities would factor in when costing care packages. This meant a shift towards shadowing: ‘we cannot afford to bring six people in to sit down at this table and do a day’s worth of digital training … so when we do training we often go out and meet with the carer whilst they are doing their care calls, we’ll shadow them and we’ll do the training alongside’. In other words, informal training also required adequate resourcing.

Interviewees also discussed a ‘learning together’ approach to technology. A manager at Clover described how, when compulsory training was shifted to an online platform, staff ‘would come to me and say, “I cannot do training online” … I said, “Not a problem, we’ll do it online and we’ll do it together”’. Shifts had to be rearranged before the care worker could go to the head office and ‘sit’ with the manager to practise. This was in part as small organisations, such as Clover, did not have specialist IT roles - meaning that the operations manager would work on ‘digital’, such as developing training materials, when they had completed their core tasks and had ‘time to play’. At the same organisation, staff members (who were now nominated to become Digital Champions) would experiment with digital solutions to simplify administrative tasks and reduce the reliance on printed forms. For example, a team leader would put up QR codes in the supported living houses that support workers could use to access Microsoft Forms. As the team members completed the form (for example, with their Covid vaccination dates) the information would automatically feed into an Excel table. This approach was strengthened through personalised support from the team leader, who explained: ‘I will always say to my team, “If you do not understand or you need somebody to do it, I’ll come and show you in person”’.

Peer support was also a key facilitator at Dill, where a care lead said ‘I’m not trained, but I know how things work so I can help them how to do that … like a peer training’. A care worker said when they need help, they would go to ‘either somebody else within my team or the care leader, because they usually know quite a lot’. Similarly a care worker at Anise described a network of peer support: ‘you can ask a colleague, you can ask your manager, you can ask the team lead’. According to a manager at the same organisation, repeated use of technology improved confidence: ‘the more they use it, the more confident… the things that you are doing on an everyday basis are just like second nature now’. Then, once people felt a bit more confident using technologies, they started to experiment and solve problems by themselves: a senior care worker at Basil said that ‘if you get stuck you can Google things, can’t you?’

### 3.3 ‘I’m here, but my phone’s dead’: beyond ‘digital skills’?

Issues in the way that technology impacted care were not always about learning and developing digital skills to engage with the functions of devices and systems, but were more about the limitations of digital systems and devices - which meant staff had to develop ‘workarounds’ to mitigate malfunctions (if possible). These issues could not be rectified by ‘upskilling’ staff, but instead relied upon their creativity and flexibility. For example, technology did not always function well with other devices or existing non-digital systems, for example between rostering and care records systems (at Fennel), or when care workers were required to use their personal phones. At Basil this impacted whether care workers were able to sign in using the NFC (near-field communication) tag: ‘I know one girl that I’ve looked at it, somebody else has looked at it, we just cannot get it working and she refuses to buy a new phone … I do not blame her, you know?’. Using paper and digital records simultaneously caused challenges for some staff. The dietician at Elderflower said:

*My brain rattles half of the time, so to try to remember I’ve got this system to use, then I need to use that system, then I’ve got a spreadsheet that’s sitting there, then I’ve got a Word document that’s sitting there. You do need something that’s more cohesive, that you are not expecting people to move all over the place with technology.*

The digital technologies in use were also contingent on adequate connectivity, with examples at Ginseng and Anise where gaps or lapses in internet connectivity, rather than the skills of the workforce, hampered the use of digital devices and systems.

There were also instances where specific devices and systems malfunctioned regardless of the skills of the care workforce, which then required staff to navigate these challenges. Technical issues impacted the systems used in the company offices, with an operations director Ginseng explaining: ‘not long ago all of our systems lost and we all lost access to our shared hard drive, so I lost all my contracts, all policies. I do not save them anywhere else, so that affected us for over a week, it was kind of like we were lost parts’. Similarly, at Clover, a team leader said, ‘technology is great when it works, and it’s also making sure you have got that backup because, actually, when I first started my laptop broke, the hard drive went and I lost everything’. The potential that systems might stop functioning meant keeping backups. Additionally, depleted phone batteries and running out of mobile data meant that staff at the domiciliary companies developed workarounds. A Care Manager at Basil said their system for recording care and logging in and out of shifts:

*Say, they run out of battery or their phone died, which does happen sometimes, I get an automatic alert through email that’ll tell me if they’re late for a call. So, I’ll check the rota and if they’ve not logged in, I’ll ring them and I’ll say, “Where are you?” And they’ll go, “I’m here, but my phone’s dead.”*

There were also examples where while the technologies did not malfunction or lack connection, they were designed in ways that their intended functions were not always appropriate for the care tasks. For example, digital care records were viewed by interviewees as more readily accessed in comparison to paper records, but these systems were not always personalised, taking more time to complete and antithetical to ‘person-centred care’. At Anise a care worker said, ‘there’s a lot more questions on their personal care and things like that, that you would not necessarily have put when you have written it down’, with some tasks (like applying makeup or shaving someone’s beard) not relevant to all the people being cared for. Simultaneously, there was not always space to provide enough information - another care worker at the same organisation was of the view that, ‘there are things that are just outlined, questions that are just there, more like a robot saying, “Yes, no, yes, no, yes, no,” Whereby maybe sometimes, I feel like you could explain further’. A care worker at Fennel described the potential ramifications of too little information being provided:

*Tick the box, provide tea, so you tick the box. Well, what did they have for tea? Because if they suddenly have an allergic reaction and they find that somebody has given them prawn curry and they’re allergic to prawns, it’s all those extra underlying things that come from that, that you don’t see.*

At Elderflower, the digital care planning system worked well for care workers and nurses but not for therapists, who could not enter detailed comments. As the Head of Therapies said: ‘it’s not designed for therapy notes and that is one of the barriers for us’. The limitations of the digital system also meant that the therapies team kept a separate online folder to record falls because they were not sure if the same fall was reported by multiple care workers in the app. At the same organisation, multiple interviewees emphasised that some assistive technologies - specialist beds - were poorly designed, as the dietician explained:

*We have profiling beds. … The bane of my life, in all honesty. … Because you’ve got a bed that’s meant to be for people who aren’t really mobile and you can’t get them in and out of bed to weigh them. But the only way you can weigh them is if you first lift them up off the bed, zero, and put them back down. So you have to essentially hoist someone off. So it kind of defeats the object of having a bed that can weigh residents.*

Difficulties related to the design of the technology extended into care planning systems, which would generate an amount of information that could be overwhelming. A staff member at Basil described how this ‘multitude of notifications’ meant that important information might be dismissed or overlooked. The care planning system in Dill likewise generated a large number of notifications for staff members - but these were for the care workers themselves. The app would notify workers if a task had not been completed but as this notification was not audible, it was more likely to be missed. At Anise, where they were also using the same ‘silent’ system, some care workers used napkins to write out a schedule for repositioning and offering fluids to the people they were caring for. One care worker questioned why the technology developer might have designed the system in such a way: ‘what’s the rationale? It would be good to have the dialogue, would not it, to find out where that came from?’. There did not, however, seem to be much dialogue between providers and technology organisations, with the result then being that staff would adapt to better accommodate the technology.

## 4 Discussion

In countries where care systems are facing issues of sustainability, many governments have proposed technology as part of the solution. In England, the increased use of digital technology in care provision has been advocated in policy discourse as a means to deliver both enhanced quality and improved efficiency. The care workforce’s ability to accommodate and utilise digital technologies has also been the subject of policy attention: alongside publishing policy documents arguing for the need for skills development, the Government has produced resources, like the ‘Adult Social Care Digital Skills Framework’ (Digitising Social Care, 2023). This paper has empirically examined these policy claims about digital skill gaps by focussing on the workforce. While the framework (and other policy documents) suggest an expansive, homogenous audience of ‘people working in adult social care who are looking to develop their skills’, we point to heterogeneity. Reflecting findings of Rolewicz et al. (2021), there is variety in organisations, roles, and technologies in the sector. For care workers, the majority of the uses for technology fit with the framework’s second category, of ‘technical skills for using technology’ - including recording care and logging in via apps, and using monitoring systems and assistive equipment. Office staff such as managers, HR staff, and those involved in commissioning and brokering (with local authorities) used other systems too, which were not necessarily care specific and sometimes extended into theme four of the framework, ‘using and managing data’. Further digital systems were used in roles such as pharmacy staff, therapy assistants, kitchen staff, housekeepers in care homes.

Alongside drawing out the diversity of technologies and roles within ‘the care workforce’, we have discussed existing skill levels among staff. In the introduction we described terminology related to skills as ‘fuzzy’, and referred to somewhat of a conflation in policy between skills and confidence. This fuzziness and conflation was apparent in perspectives of our interviewees, with skills often discussed in terms of confidence in using devices and systems, and comfortability with completing digital tasks [reiterating the findings of Blake et al. (2021)]. Seeing confidence as thus an aspect of skill, we explored the assumptions - or expectations - that the workforce is low in confidence, ‘techno-phobic’ and or/ in need of ‘modernisation’. As discussed in the first subsection of results, some staff members were initially reluctant or trepidatious, yet they adapted and learnt to use the technologies they needed in their everyday work. Ease of learning was, however, mediated by earlier experience of using digital technologies such as smartphones, which was linked to age and English language skills. While such factors could impact the amount of time that it took workers to adapt, we found that all care workers described eventually gaining confidence and reaching a level of comfortability with the digital systems - the most challenging technology was described as the more ‘mainstream’ tools such as Excel, typically used by staff working in offices and in management roles.

Facilitators to the development of skills and confidence ranged from formal training to informal support. In terms of formal training, we found organisation and job-specific workplace training was important as digital technologies and devices can quickly become obsolete, and care providers often need to introduce new technologies while discontinuing the use of others. This training needed to be provided frequently because staff turnover was high - and prior experience in social care did not always mean that new staff would be confident with digital systems, as they often had to learn to use new systems and devices. Formal vocational training for care workers discussed in policy might not keep pace with this variation in systems within organisations, or with the speed of change in adopting new systems. As such, a policy priority should be financially and strategically supporting internal, on the job learning and development (e.g., having trainers on site, and putting in place training programmes tailored to the needs of workers at a particular organisation following an assessment of existing digital skills) rather than funding external training. Understanding skill requirements could be supported at a sectoral level alongside trade unions, e.g., through the proposed Adult Social Care Negotiating Body (UK Government, 2024) - some unions in the sector also provide digital training for members (UNISON, n.d.).

As well as formal training, our findings emphasise the importance of informal ‘learning by doing’ and peer support as facilitators to skill development [reflecting findings of Lloyd and Payne (2023)]. A supportive environment where workers could ask questions and admit errors without fear was important too, and opportunities to use digital technology at work then improved workers’ self-assessed confidence levels. However, these opportunities depended on capacity, for example time to practice with devices or shadow other staff. The focus on peer support was also impacted by, and further impacted, capacity levels in an overworked and understaffed workforce. While some new training and management roles had been created at the case study organisations (e.g., digital lead, digital care lead), staff who played a central role in peer support (‘super users’ or ‘Digital Champions’) did so in addition to their ‘day jobs’. It thus contributes to the invisible, additional labour which has been found to be characteristic of care contexts (Hamblin, 2022; Hamblin et al., 2023), and creates issues of sustainability, given the reliance on the individuals providing peer support. There was also an apparent lack of enhanced pay associated with these positions, and instead it was assumed - or hoped - that these staff would enjoy helping others with technology. As noted by Oung et al. (2021), digital skills need to be situated within a ‘clear pay and progression framework’ and are undermined by limited career progression for care workers. Despite this evidence the proposed ‘Care Workforce Pathway for adult social care’ (Department for Health and Social Care, 2024b) - which followed the ‘Adult Social Care Digital Skills Framework’(Digitising Social Care, 2023) - does not connect progression with pay increases.

Our findings also illustrate how difficulties with using technology relate to the design of the technology itself. The ‘Adult Social Care Digital Skills Framework’ (Digitising Social Care, 2023) includes ‘Solve basic problems when using technology’, but often issues encountered by the care workforce were more fundamental than ‘basic’. Recommended actions such as updating software, ‘Following instructions set out in your organisation’s policies and procedures’, ‘Using the device or software manual to help you solve problems, ‘Using the internet, chat facilities or technical support helplines’ and ‘Using online tutorials, FAQs and advice forums’ were not sufficient to manage these challenges, and therefore the care workforce utilised skills beyond those laid out in the framework. Care workers had the technical skills to use new devices and systems, but the technologies were not necessarily inherently ‘user-friendly’ in the tasks they engendered. Technologies were not always tailored perfectly to the work processes of a particular organisation either, instead requiring that workers actively adapt their work practices around the technologies. Workers thus not only learnt to use new technologies, but adapted skills in other areas.

Importantly, our findings illustrate how workers would critique the technologies in ways which were informed by their care skills, highlighting why certain equipment and apps might be unsuitable for care tasks and suggesting a point of tension between the perspectives of those designing devices and systems and those using them in the provision of care. Running counter to the expectation that technology will improve personalisation, platforms used for recording care could also involve generic tick lists of tasks and extraneous details which are not always relevant to the care provided. Sometimes the unsuitability of technology was related to it malfunctioning. This not only created frustration, but further added to the workload of individual members of staff who had to work around the problems. At an organisational level, some managers then explained to the technology companies what the problems were, and tried different ‘patches’ and updates to solve them. A particular obstacle discussed by participants was the lack of integration or interoperability between digital systems, sometimes due to technologies not ‘talking to each other’ well (requiring workarounds to facilitate communication between different devices and systems). Identifying these obstacles related to technology functionality requires, as with identifying where training is needed, listening to staff. Given their understanding of issues around technology alongside work processes, care workers are ideally placed to provide feedback about the suitability and functionality of systems and devices. It is therefore important for developers - and those procuring and selecting technology, e.g., the senior leadership of care providers and staff at local authorities to take the experiences of care workers into account, and for feedback mechanisms to be in place to facilitate the ‘coproduction’ of digital solutions (Department for Health and Social Care, 2023).

### 4.1 Limitations

In making these arguments, our study has a number of limitations. The first is that it is based on a small number of case studies in England: conducting additional case studies would enable more systematic comparison between different care settings and types of service providers. Further research could also focus on digital skills of the social care workforce in all of the four home nations of the UK - allowing the studies to consider the effects of different policy approaches to skills development, with compulsory training and registration (‘professionalisation’) everywhere except in England. Further studies could also benefit from incorporating the views and experiences on digital technologies in social care of those using care services and their family members.

### 4.2 Conclusion

This paper has critically examined how current policy strategies related to digital skills connect to embedded practices of social care workplaces. Digital technology is seen as an innovative way to resolve the perceived ‘crisis’ in adult social care, with policy discourse - for instance, the ambitiously titled policy paper ‘Data Saves Lives’ - espousing the benefits of increased data collection and digitalisation of care records. While technology is positioned as the ‘opportunity’ in the policy discourse, the workforce is positioned as the ‘challenge’. We have problematised this discourse by showing empirically that the workforce is able to adapt to new and fast changing digital technologies at work. There is therefore a disconnect between the policy focus on skills and the need for skill development. Devices and systems being integrated into care work did not require significantly enhanced skills related to technology, but did require time, opportunity, and peer support (with some groups requiring more support than others). Learning and teaching digital skills in social care were thus ongoing processes, rather than a one off fix. We also found that the design of the technology itself can limit its adoption, and that workers adapt and craft technologies to serve care purposes. A consequent implication for practice and policy is that a grounded approach to the design and implementation of technology where care workers’ practice-based experiences and perspectives are considered is needed. Overall, we have demonstrated that the diversity in the use of digital technologies, digital skills, job roles, and care settings, make initiatives like a digital skills framework potentially difficult to implement. This is combined with the wider difficulty of enacting any kind of standardised training and qualifications in a fragmented sector (other than that which is statutorily required). Discussions about digital skills cannot ignore this context of fragmentation and marketisation in England and globally, and the effects that care workforce shortages and high turnover - connected to low pay and poor terms and conditions of employment - continue to have on the processes of both formal and informal skill development.

## Data Availability

The datasets presented in this article are not readily available because they are part of an ongoing project, and will be uploaded to a data archive at a later date. Requests to access the datasets should be directed to the corresponding author.
